# Estimation of the Cognitive Functioning of the Elderly by AI Agents: A Comparative Analysis of the Effects of the Psychological Burden of Intervention

**DOI:** 10.3390/healthcare12181821

**Published:** 2024-09-11

**Authors:** Toshiharu Igarashi, Katsuya Iijima, Kunio Nitta, Yu Chen

**Affiliations:** 1 Simulation of Complex Systems Laboratory, Department of Human and Engineered Environmental Studies, Graduate School of Frontier Sciences, The University of Tokyo, Tokyo 277-8563, Japan; 2 AI-UX Design Research Institution, Advanced Institute of Industrial Technology, 10-40 Higashi-Oi 1-Chome, Shinagawa, Tokyo 140-0011, Japan; 3 Institute of Gerontology (IOG), The University of Tokyo, Tokyo 113-8656, Japan; 4 Institute for Future Initiatives (IFI), The University of Tokyo, Tokyo 113-0033, Japan; 5 Tsukushikai Medical Corporation, Tokyo 186-0005, Japan

**Keywords:** AI agents, cognitive function estimation, Alzheimer’s disease, dementia, psychological burden

## Abstract

In recent years, an increasing number of studies have begun to use conversational data in spontaneous speech to estimate cognitive function in older people. The targets of spontaneous speech with older people used to be physicians and licensed psychologists, but it is now possible to have conversations with fully automatic AI agents. However, it has not yet been clarified what difference there is in conversational communication with older people when the examiner is a human or an AI agent. This study explored the psychological burden experienced by elderly participants during cognitive function assessments, comparing interactions with human and AI conversational partners. Thirty-four participants, averaging 78.71 years of age, were evaluated using the Mini-Mental State Examination (MMSE), the Visual Analogue Scale (VAS), and the State-Trait Anxiety Inventory (STAI). The objective was to assess the psychological impact of different conversational formats on the participants. The results indicated that the mental strain, as measured by VAS and STAI scores, was significantly higher during the MMSE sessions compared to other conversational interactions (*p* < 0.01). Notably, there was no significant difference in the mental burden between conversations with humans and AI agents, suggesting that AI-based systems could be as effective as human interaction in cognitive assessments.

## 1. Introduction

Life expectancy is increasing globally. According to the World Health Statistics 2023 of the World Health Organization (WHO), the global average life expectancy for men and women was 73.3 years in 2019 [1]. Advances in medical technology and improvements in lifestyle led to an increase in the global average life expectancy from 67.9 years in the early 1990s to 73.4 years in 2021, an increase of 5.5 years (about 6%) over 20 years. By 2048, life expectancy is expected to reach 77.0 years globally and 82.4 years in the Western Pacific region, including Japan.

The rise in global life expectancy has also created new challenges for aging societies. The gap between life expectancy and healthy life expectancy is a more pressing issue in aging societies, as the increase in healthy life expectancy has lagged behind the rise in life expectancy. The WHO notes that while life expectancy has increased due to reduced mortality, it does not equate to a decrease in the years spent living with disability. In Japan, the Ministry of Health, Labour, and Welfare (MHLW) stresses the need to extend healthy life expectancy, which is the duration during which people can maintain essential functions for social life and live without daily restrictions due to health issues [2]. Among age-related disease measures, efforts to combat cognitive impairment have garnered particular attention. Around 50 million people worldwide suffer from cognitive impairment, and this number is projected to rise to approximately 152 million by 2050 [3]. Cognitive impairment leads to a loss of independence, an increased caregiving burden, and heightened economic costs. In Japan, the social cost of cognitive impairment was significant, amounting to 14.263 trillion yen in 2014 [4]. Early detection and treatment promotion will optimize the use of limited financial resources.

According to the “Guidelines for the Diagnosis and Treatment of Dementia” (Japan Neurological Society) [5], cognitive dysfunction is defined as “a generalized decline in intellectual functions once acquired due to acquired brain dysfunction that interferes with social and daily life, occurring in the absence of disturbances in consciousness”. Intellectual functions include memory, orientation, language, recognition, calculation, thinking, motivation, and judgment, which are crucial for planning and performing daily activities such as cleaning, doing laundry, eating, and going out. As a result, cognitive dysfunction may cause difficulties in daily planning and activities, posing serious challenges to daily life [6,7]. Impaired executive function due to cognitive dysfunction may hinder self-care, potentially compromising physical health. Moreover, certain diseases, such as diabetes, are linked to cognitive dysfunction [8,9]. Additionally, cognitive dysfunction may prolong hospitalization [10,11]. The condition brings diverse effects, including reduced patient quality of life, longer hospital stays, higher mortality, and elevated social costs. Effective treatments for cognitive dysfunction have not been established. However, depending on the cause and condition, appropriate support and treatment can delay disease progression. Thus, early detection and intervention are crucial.

Many cognitive function tests to diagnose cognitive dysfunction raise issues related to the testing time, invasiveness, psychological burden on patients, and cost of necessary equipment and facilities. CT and MRI tests can be performed only in large hospitals that have such facilities and are likely to cause a testing time and cost burden and invasiveness to patients. In neuropsychological testing, while some tests can be performed in a relatively short time, they must be performed by a clinical psychologist with specialized knowledge, and the results may be affected by the patient’s physical condition. The HDS-R (Hasegawa’s Dementia Scale-Revised) [12] and FAST (Functional Assessment Staging of Alzheimer’s Disease) [13] are examples of cognitive function assessment scales. However, the HDS-R requires careful consideration because differences in social backgrounds, such as the years of education and living environments of the elderly, may have emerged between the past and present, potentially affecting test performance [14]. Additionally, FAST is not an interactive test but an assessment based on interviews with family members and caregivers. On the other hand, MMSE [15] has been used across a variety of patients, including cancer patients and stroke survivors [16,17,18], as it is well-accepted by medical professionals working with patients with cognitive impairment. MMSE has a 70% recognition rate in the field of geriatric psychiatry in Japan [19]. Although MMSE scores have often been reported to be influenced by the number of years of education of the examinees [20], these studies were all conducted outside of Japan. Current reports both in Japan and abroad indicate that MMSE scores are not significantly influenced by the patient’s years of education [14,21,22].

In cognitive function testing, it is essential to reduce the burden on the elderly and perform the tests at a low cost. The use of AI in clinical settings to support the elderly is actively studied and discussed today, and its usefulness and challenges are being explored.

In this study, we overviewed the early detection of cognitive impairment using previous studies with an overview of cognitive testing methods. We also summarized some previous studies using virtual agents. Then, we analyzed the psychological burden on the elderly when the Mini-Mental State Examination (MMSE) was administered to 34 elderly subjects by a human examiner or an AI agent as an administrator.

This study may provide a new perspective on the problems that many conventional cognitive function tests for diagnosing cognitive dysfunction have regarding the testing time, invasiveness, patient psychological burden, and cost of necessary equipment and facilities.

## 2. Related Works

### 2.1. Significance of Early Detection of Cognitive Impairment

Early detection of cognitive impairment can contribute to extending healthy life expectancy. Cognitive dysfunction may hinder executive function, complicating self-care and impacting physical health. Additionally, cognitive impairment has been associated with certain diseases. For example, older diabetic patients exhibit lower executive function compared to non-diabetic older adults, which is linked to reduced executive function and, consequently, the increased use of healthcare services [8]. In chronic obstructive pulmonary disease, mortality is higher in patients with cognitive impairment than in those without, and cognitive impairment significantly raises the likelihood of severe sepsis and death [9]. Additionally, cognitive dysfunction may lengthen hospital stays [10,11].

The early detection and treatment of cognitive impairment can help reduce social costs. As the number of patients with cognitive impairment grows, so does the economic burden on society. Studies in various countries have already estimated the social costs of cognitive impairment.

In the United States, the total cost of caring for cognitively impaired patients in 2010 was estimated to be between USD 159 billion and USD 215 billion. These costs are expected to double by 2040 [23]. In Ireland, the estimated economic and social cost of addressing cognitive impairment in 2010 was EUR 1.69 billion per year, indicating that future support systems for cognitive impairment will struggle without a significant increase in resources to meet these costs [24]. According to a 2010 global study, the estimated total global cost of dementia was US$604 billion [25].

In Japan, the medical costs related to cognitive impairment are estimated at JPY 1911.4 billion for the fiscal year 2014. Long-term care costs are estimated at JPY 6444.1 billion, and informal care costs at JPY 6158.4 billion, resulting in a total social cost of cognitive impairment of JPY 14.514 trillion in 2014. It is estimated to reach JPY 24.263 trillion by 2060 [4]. Therefore, from a social cost perspective, there is an urgent need for the research and development of cost-effective methods for the early detection and treatment of cognitive impairment.

The early detection of cognitive dysfunction allows the patient’s right to self-determination in assets and care to be respected [26]. Even without established treatments for cognitive dysfunction, the patient’s preferences can be considered when planning medical and nursing care.

It is also important that a proxy is appointed for a patient with cognitive impairment while the individual still has sufficient cognitive ability [26].

The early detection of cognitive impairment when the person still has sufficient cognitive ability allows for the respect of basic rights such as self-determination and privacy.

On the other hand, approaches to the early detection of cognitive dysfunction carry risks. These include the potential loss of status previously attained due to health, a decrease in reputation, and the possibility of depressive reactions [27].

The very act of undergoing a cognitive impairment test can lead patients to feel their abilities are being doubted, causing a psychological burden and distress.

### 2.2. Testing for Cognitive Impairment

The diagnosis of cognitive dysfunction typically involves a combination of imaging and neuropsychological tests. In particular, data from functional brain imaging tests that noninvasively and precisely examine regional brain activation are important. For example, positron emission tomography (PET) and magnetic resonance imaging (MRI) diagnose qualitative brain changes through imaging, but these methods are time-consuming and require expensive equipment [28,29]. Psychological assessments are conducted to measure an individual’s behavior, emotions, or cognitive functions. According to a study by Miller et al., these tests can potentially induce stress and anxiety in examinees. For instance, because the results of psychological assessments are often used for significant decision-making, examinees may feel pressured [30]. Additionally, the American Psychological Association (APA) has addressed the burden on examinees during psychological testing. The APA guidelines highlight the potential negative impact of conducting psychological tests, assessments, and measurements, particularly in high-pressure or uncertain situations where the outcomes may significantly affect an individual’s future [31]. Neuropsychological tests assess higher brain functions and are essential for treating cognitive impairment [32]. Specifically, the Mini-Mental State Examination (MMSE) and the Hasegawa Dementia Scale (HDS-R) are used for quick and convenient screening.

However, the questions on the test forms are fixed, which can lead patients to memorize them, making them unsuitable for periodic monitoring [16]. Additionally, being tested for cognitive function can make test patients feel that their abilities are being doubted, causing a mental burden and distress. In fact, many elderly individuals refuse testing for cognitive impairment, and it has been reported that 16% of patients with Alzheimer’s disease exhibit catastrophic reactions such as anxiety, anger, and refusal during testing [33]. In addition, various factors such as the way the examiner asks questions and their attitude may influence the results of cognitive function tests, so it is recommended that the tests be administered by a person with specialized training. Additionally, Mehrabian et al. have pointed out that in communication, factors obtained from the face carry about 3/2 the weight of those obtained from the voice in inferring the speaker’s attitude [34].

Methods that are unaffected by some of the problems found in interactive cognitive function tests, such as the Clinical Dementia Rating (CDR) and the N-Type Mental Status Scale for the Elderly (NM Scale), which are observational assessments, have been used [35]. While these methods have the advantage of not placing a burden on the elderly, they lack objectivity compared to interactive assessments, and the results may vary depending on the assessor’s knowledge and experience. A study by Kawaguchi and Sato showed that caregivers in nursing homes do not have an accurate grasp of the cognitive functions of the cognitively impaired elderly people they care for on a daily basis [36].

In recent years, screening for cognitive dysfunction based on the elderly’s language ability in spontaneous speech has been gaining attention. This type of screening is non-invasive and does not require prolonged stays in medical facilities. Regular monitoring of cognitive function is expected to lead to the early detection of cognitive dysfunction. Developing an objective method for using daily conversation as a screening tool for detecting cognitive decline is also expected to reduce the cost of visits for patients, caregivers, and healthcare providers during testing. Various methods may be used to elicit natural speech, such as home-visit support and telephone support, but in the past few years, AI agents and robots have garnered particular attention. Conversational AI agents are among the many digital technologies being implemented in healthcare to improve the availability of healthcare services and address current healthcare challenges such as the shortage of healthcare providers, which impacts accessibility [37,38].

### 2.3. Tests Using Language Skills

There are a variety of previous studies using different approaches to assess cognitive function and identify impairment at an early stage. For example, some studies have used verbal and image data to assess cognitive function. Liu et al. used conversational and imagery data obtained from clinical trials to examine the potential for cognitive function improvement. They compared an experimental group that frequently engaged in semi-structured conversations with a control group that only received weekly telephone checks, analyzing linguistic markers and image features. This study represents a new approach to assessing cognitive function by combining both verbal and imagery data [39].

Studies conducted without actual human participants include a study by Yoshii et al. that attempted to screen for mild cognitive impairment (MCI) through conversations with a humanoid robot [40], a study by Liang et al. that evaluated cognitive impairment detection using voice assistant commands [41], and Agbavor and Liang’s study [42] that used an automatic speech recognition model Wav2Vec 2.0 to convert speech to text and captured lexical, syntactic, and semantic properties with GPT-3. Those focusing on speech and language data include a study by Hamrick et al. that analyzed lexical features to discriminate the presence of cognitive impairment from the spontaneous speech of elderly people, computing 16 lexical and semantic features from speech [43], and a study by Tang et al. that investigated the early identification of cognitive function through weekly phone checks and video chats [44].

These previous studies demonstrate a variety of methodologies and approaches in the assessment of cognitive function and its early identification, including some that use voice assistants and robots. However, there is still a lack of quantitative analysis on how much methods for estimating cognitive function using daily conversation actually reduce the burden on patients, and what differences occur when the target of the conversation is a real human versus an AI agent. This study differs from the approaches of the above-mentioned previous studies in that it compares the psychological impact on elderly patients undergoing tests when the examiner is either a human or an AI agent, as used in actual clinical practice.

### 2.4. Support for the Elderly Using Virtual Agents

Conversational virtual agents use computer-simulated virtual characters as so-called avatars, which can range from simple cartoon-like characters to highly detailed three-dimensional forms and resemble humans or other entities [38]. These agents interact with humans on mobile, web-based, or audio-based platforms using machine learning or natural language processing. In an overseas study conducted in the UK, a group with mild cognitive impairment and a healthy control group interacted with a virtual agent using pre-recorded questions. It was found that the group with mild cognitive impairment had shorter speech times compared to the healthy control group, suggesting that speech analysis may contribute to diagnosing patients with cognitive impairment [39].

Another study aimed to establish the requirements for voice interfaces for AI assistants by focusing on dementia patients, caregivers, and elderly people without dementia. For people with dementia, AI assistants should act as guides to encourage patients, while for elderly people without dementia, assistance should be to the point and not excessive. This indicates that the requirements vary depending on the user’s condition [45]. In Japan, NTT Communications Corporation launched the operation of the “Brain Health Check Toll Free Number” in 2022 [46]. This service allows AI to measure changes in cognitive function based on speech content and voice quality in a 20-s utterance, enabling users to check their brain health discreetly. A study conducted on a group of patients with cognitive dysfunction (MMSE average of 12.2) showed that conversations between the patient and the agent were successful, with the agent being received favorably. The study also found that speech volume increased when the agent provided topics of interest to the participant, suggesting the effectiveness of dialogue with an animated agent for patients with cognitive impairment [47].

In research using robots, several surveys and studies have been conducted using Papero-i [48], developed by the NEC Corporation, including a study by Igarashi et al. that demonstrated self-disclosure by a robot significantly improved the quantity and quality of dialogue among elderly individuals, and a study by Kobayashi et al. that used Papero-i for dementia diagnosis based on natural conversations with elderly people living alone. If there is a suspicion of dementia, the system notifies distant family members via social media. This early detection system for dementia was shown to be effective [49].

Recent comparative studies have also examined the differences between human–human communication and human–AI communication.

For example, a study by Hill et al. (2015) found that conversations with chatbots tend to be longer; however, the messages were shorter and had less lexical diversity compared to human–human interactions [50].

Additionally, a study by Mou et al. (2017) indicated that when interacting with AI, human users exhibit lower levels of openness, agreeableness, extraversion, conscientiousness, and self-disclosure compared to conversations with humans, highlighting different personality traits and communication attributes in human–AI interactions [51]. Hewitt and Beaver (2020) compared communication styles between customer service agents and virtual agents, noting that the quantity and quality of communication can differ significantly between human–human interactions and human–AI interactions [52]. Furthermore, Diederich et al. (2022) reviewed multiple studies on conversational agents. They highlighted that, due to advances in natural language processing, conversational agents are gaining increasing attention in both academia and practice. The authors emphasized the importance of designing conversational agents to meet their intended purposes and with a better understanding of how humans interact with them. They also identified the need to accommodate diverse user groups, such as individuals with disabilities and the elderly, as a future challenge [53].

These previous studies highlight the evolving nature of communication between humans and AI, underscoring the need for further research to optimize AI-based interactions. While the use of AI agents and robots is expected to reduce costs, particularly for healthcare professionals and caregivers conducting examinations, there has been no confirmed difference in patients’ psychological resistance to examinations compared to human examiners. This study aims to contribute new insights into the nature of communication between humans and AI through a comparative study involving elderly individuals, a diverse user group, and their interactions with both human and AI interlocutors.

### 2.5. Contribution of This Study

The early detection of cognitive impairment affects extending the healthy life expectancy of the elderly and reducing social costs, so it is desirable to promptly conduct cognitive function tests when cognitive impairment is suspected. Although the research and development of cost-effective approaches for early detection and treatment are expected to lead to a reduction in future social costs, the high burden on participants in cognitive function tests and the lack of empirical studies on designing voice interfaces for the elderly underscore the need for the research and consideration of alternative approaches to conventional methods. For example, AI agents may be less restricted by the location of the test, while human test administrators generally need to travel to the test site for face-to-face testing. AI agents may be perceived by users as unbiased, and when discussing personal concerns with AI agents, they may help identify factors that could reduce the time and financial costs of training and deploying practitioners, enable testing in the absence of professionals, or decrease the burden on participants. It has also been suggested that users may experience less anxiety when discussing personal concerns with an AI agent compared to a human [54].

Psychological assessments are conducted to measure an individual’s behavior, emotions, and cognitive functions. However, these tests can potentially induce stress and anxiety in examinees, as the results are often used for significant decision-making that can impact the examinee’s future [30,31]. Since cognitive function evaluations can greatly affect an individual’s future employment, living conditions, and care, the pressure and psychological burden on examinees are considerable.

Currently, research is being conducted to estimate cognitive function using everyday conversation and leverage AI to reduce the burden on examinees. However, there are still insufficient quantitative analyses on how much this burden is actually reduced and what differences arise when the conversation involves a human versus an AI agent.

Recent comparative studies have explored the differences between human–human communication and human–AI communication. For example, Hill et al. (2015) found that conversations with AI chatbots tend to be longer, but the messages are shorter and exhibit less lexical diversity compared to human–human interactions [50].

Additionally, Silkej (2020) found that in text-based communication with students, the number of words per message was lower when interacting with a chatbot compared to conversations with humans [55].

However, these studies do not cover diverse user groups. For example, they do not account for differences in cognitive function, and it remains unclear whether the same results would be obtained with varying levels of cognitive ability. Future challenges in conversational agent research include accommodating diverse user groups, such as individuals with disabilities and the elderly, reflecting the importance of conducting comparative studies between human and AI interactions across different cognitive function groups [53].

This study aims to contribute to the examination of psychological anxiety and pressure in cognitive function assessments, which can significantly impact an individual’s future. By implementing conversational agents that cater to diverse user groups and comparing AI-based and human-based interactions, the study explores the potential of these conversations to reduce the psychological burden and proposes new methods for cognitive function evaluation.

## 3. Methods

In this study, ECG measurements were taken under three different situations: during MMSE, during a daily conversation with a human, and during a daily conversation via AI. The experiment was conducted at the Yagawa Day Service Center and the Kunitachi Silver Human Resource Center in Kunitachi City, Tokyo. Nineteen participants participated at each facility, with either a human or AI acting as the questioner. The impact of different dialogue partners on the psychological burden of the participants was compared.

### 3.1. Participants

A total of 34 elderly persons (12 men and 22 women) were included in the study; they were elderly persons living in the community who were not in institutions, etc. Of the 34, 17 were day service users and 17 belonged to the Silver Human Resource Center in the same area. To ensure that the results were not affected by dialect, we selected those residing in Tokyo, Japan. The number of participants was limited to those who did not have any disease that would affect their listening or speech in general conversation, such as hearing loss, for example. Initially, 19 participants were included in each group, but 2 participants from each group died or were transferred to other hospitals during the study period and therefore could not be followed up. The participants were those who consented to the purpose of the study. For elderly patients with cognitive decline, their families were informed and gave consent to the study.

### 3.2. Obtained Data

In this study, several rating scales and measures were used to assess differences in the impact on cognitive function and mental strain among older adults. The Mini-Mental State Examination (MMSE) [15] was used to measure cognitive function. This test takes 6–10 min to complete, consists of 11 items, and assesses orientation, memory, calculation, language, and visuospatial abilities for a total score of 30 points; a score of 23 or less indicates a suspicion of dementia, and a score of 27 or less indicates mild cognitive impairment (MCI).

In Japan, a Japanese version (MMSE-J) has been created by several translators and is frequently used as an auxiliary tool for the diagnosis of cognitive impairment. In this study, we decided to adopt the internationally used MMSE and used the Sugimoto translation of the MMSE-J [17].

The State-Trait Anxiety Inventory (STAI) [56] was used to measure the participants’ anxiety levels, and a translated version of the new STAI [57] was used. Participants respond to each question on a four-point scale ranging from “not at all” to “very much”. The test item measures two aspects: state anxiety, which assesses transient situational reactions to how one is feeling at the moment, and trait anxiety, which assesses the tendency to react based on general emotions. The Visual Analogue Scale (VAS) [58] was used to measure the degree of mental burden in each task; the VAS is a visual rating scale and requires participants to mark the intensity of the pain or burden that they experience. For example, for pain, participants mark on a 10 cm line, with the left end representing “no pain at all” (0) and the right end representing “most pain” (100).

To consider the impact of depression on conversation, the Geriatric Depression Scale (GDS-15) [59] was used; the GDS-15 consists of 15 questions to be answered with “yes” or “no”, with a score of 5 or higher indicating a depressive tendency and a score of 10 or higher indicating depression. In addition, to collect and examine physical parameters, changes in heart rate and pulse were recorded using a Holter electrocardiograph. This allows for the analysis of levels of excitement and stress. In this study, participants wore a DigitalWalk-700 Holter electrocardiograph to collect ECG data.

### 3.3. Display Environment for AI Agents

**Figure 1:**
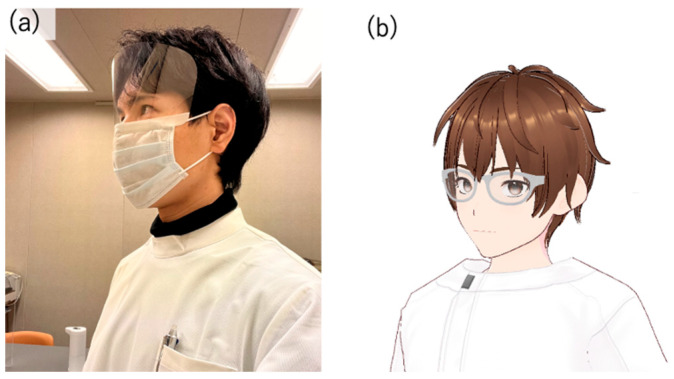
(**a**) the appearance of the author who conducted the interpersonal conversation and (**b**) the appearance of the AI agent, modeled based on the author’s appearance.

Several modules were used to implement the virtual agent, an avatar with AI dialogue capabilities. First, since the author conducted interpersonal conversations, the AI avatar’s design was made to resemble the author’s appearance (Figure 1). To achieve a design similar to the author, VRoid Studio (https://vroid.com) [60] was used for modeling. This was to minimize the impact of the avatar’s appearance on the test results, as using an avatar that looks significantly different from the person the participants assumed would be speaking could influence the results, due to individual differences in impression.

To display the designed model in a browser, we used @pixiv/three-vrm [61]. @pixiv/three-vrm is a library for loading and displaying VRM, a format for handling humanoid 3D avatar model data using three.js, on a browser, and is open-sourced by Pixiv. The AI agent was displayed on the screen of a MacBook Pro. Additionally, the background of the character incorporated the background of the room in the facility where the dialogue was taking place, creating an environment where the user felt like they were conversing with a real person.

For generating the AI agent’s speech in daily conversations, we used the Koeiro API [62]. We designed the AI agent’s mouth to move in sync with the speech using lip-sync functionality. For recognizing the user’s responses during the conversation, we employed the Web Speech API (Speech Recognition) [63]. Since speech recognition can be difficult when the timing of the agent’s speech overlaps with the participant’s, we implemented a system where the participant wears a pin microphone, and the system only recognizes speech while a button is pressed.

### 3.4. Protocols in Conversation Design

**Figure 2:**
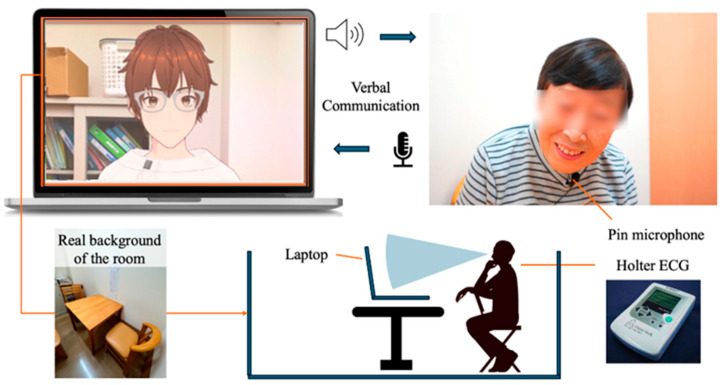
Protocol of daily conversation system for cognitive function estimation by AI agents.

**Figure 3:**
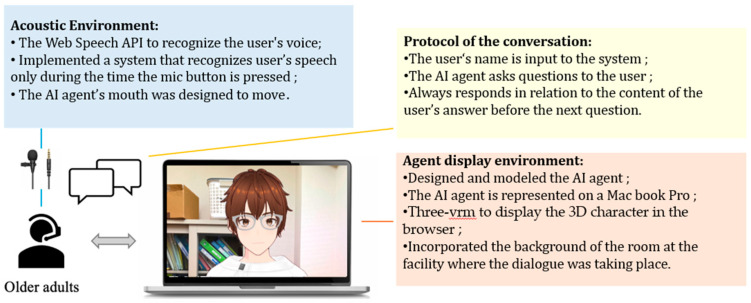
Configuration diagram showing the entire system.

The conversation design protocol was structured using a semi-structured interview technique with fixed question content. In semi-structured interviews, the examiner asks questions while adjusting their responses based on the user’s answers. If the user does not receive any reaction to their answers, there is a risk that the quality of their responses may decrease, or they may disengage from the conversation. Therefore, the conversation protocol was designed so that the AI agent always responds to the user’s answer with a related response before moving on to the next question. The implementation of dialogues, including responses corresponding to the user’s answers, was carried out using the ChatGPT API [64] (Figure 2 and Figure 3).

The user was seated in front of a PC displaying the AI agent and equipped with a pin microphone for speech recognition. Additionally, the design called for the conversation to begin with the user wearing a Holter electrocardiograph.

## 4. Evaluation

### 4.1. Implementation Procedure

In this study, an experimental approach was used to evaluate the psychological burden of tests on participants and its effect on their cognitive function. The purpose of the experiment was to visualize the psychological burden through changes in participants self-administered psychological tests and electrocardiograms under different interactive environments. This study was conducted with the approval of the ethical review boards of the University of Tokyo and the Tokyo Metropolitan Institute of Technology.

### 4.2. Hearing Items in Daily Conversation

An important previous study by Igarashi et al. on the estimation methods of cognitive functions [65] is important. In that study, generalized interview items were developed to estimate cognitive function through daily conversation. From intake interviews conducted by psychologists at hospitals, the questionnaire items were designed to ascertain patients’ abilities related to family history, physical condition, interests and concerns, ways of spending the day, memory, time-registration, and place-registration, and as a validation, practical supervision was also conducted by five licensed psychologists working at the hospitals. These were refined, and a total of 30 questions were designed as interview items for the present study, grouped into six categories: time-registration, place-registration, family history, how they spend their day, physical condition, and interests/concerns (Table 1).

### 4.3. Data Acquisition Flow

Each participant underwent three tests: a cognitive function test using the Mini-Mental State Examination (MMSE), a daily conversation with a human, and a daily conversation via AI. First, the STAI (State-Trait Anxiety Inventory) was used to measure the participants’ current and usual levels of anxiety. Then, the VAS (Visual Analogue Scale) was used to assess the participants’ self-evaluation of “how much mental strain they are currently feeling”.

Before each session of the experiment, participants were instructed to rest with their eyes open for 5 min to ensure that their heart rate was stable. Then, participants engaged in a daily conversation for 30 min. After the conversation, the mental strain during the session was again assessed using the STAI and VAS. Next, a 15-min cognitive function test using the MMSE was administered, and the STAI and VAS were used to assess the participant’s mental strain. To avoid the order of questions in the daily conversation affecting the participant’s response performance, all the questions were asked in a consistent order. Interaction tests with the AI agents were conducted at least one month after the in-person interaction tests.

## 5. Results

A total of 34 elderly persons (12 males and 22 females) were included in the study, 17 of whom were day service users and 17 of whom belonged to the Silver Human Resource Center in the same area. Initially, 19 participants were included in each group, but 2 participants from each group died or were transferred to other hospitals during the study period and therefore could not be followed up. The participants were those who consented to the purpose of the study. For elderly patients with cognitive decline, their families were informed and gave consent to the study.

The mean age was 78.71 years (SD = 6.77). The mean score of the MMSE (Mini-Mental State Examination) was 21.09 (SD = 8.26), and the mean score of the GDS (Geriatric Depression Scale) was 2.48 (SD = 2.45). The mean heart rate during each test was 75.3 (SD = 10.44) after the daily conversation with a human, and 74.8 (SD = 11.41) after the test with an AI agent. The mean heart rate after the MMSE was 74.8 (SD = 11.13).

**Figure 4:**
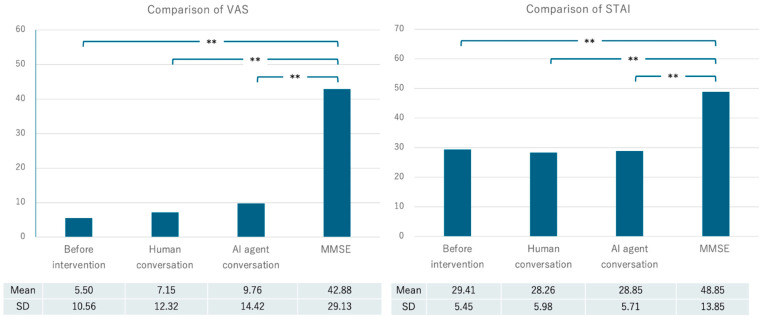
Comparison of differences in means by VAS and STAI. **: Significant differences were found at the 1% level.

The mean Visual Analogue Scale (VAS) score at the beginning of the experiment was 5.50 (SD = 10.56), the mean VAS score after the daily conversation with a human was 7.15 (SD = 12.32), and the mean VAS score after the test with the AI agent was 9.76 (SD = 14.42). The mean VAS score after the MMSE was 42.88 (SD = 29.13). The mean current state anxiety score measured by the STAI (State-Trait Anxiety Inventory) was 29.41 (SD = 5.45), the mean STAI score after the daily conversation with a human was 28.26 (SD = 5.98), and the mean score after implementation with the AI agent was 28.85 (SD = 5.71). The mean STAI score after MMSE implementation was 48.85 (SD = 13.85) (Table 2, Figure 4).

As a supplementary note, it should be mentioned that during the acquisition of ECG data, there were instances where participants with cognitive decline made contact with the ECG equipment during the conversational experiments. The equipment used was a Holter electrocardiograph, prioritizing the minimization of the burden on the participants due to its lightweight and portable nature. However, it should be noted that while this device reduces the burden on participants, it may result in slightly less stable data compared to standard equipment. To avoid psychological effects caused by an unfamiliar environment, the study was conducted in familiar surroundings rather than requiring participants to visit a hospital. This approach was achieved by using portable equipment, allowing the study to take place in a room within a familiar facility.

## 6. Analysis

### 6.1. Analysis of VAS

The Visual Analogue Scale (VAS) was used to assess the mental burden of the participants. This is a visual analogue scale that participants use to mark the intensity of pain or burden they feel. In this study, participants were asked to indicate a point on a line where the left end represents “no burden at all” (0) and the right end represents “maximum burden” (100), corresponding to their current mental burden. This method allowed the visualization and quantification of the burden experienced by participants in each section. Friedman tests were conducted in Microsoft Excel (Version 2404) on the VAS scores for 34 participants in the pre-interview condition, after the in-person conversation, after the conversation with the AI agent, and after the MMSE. The *p*-value was 1.5 × 10^−11^, indicating that the VAS scores changed with the test administration. The two post-hoc groups were tested using Bonferroni’s adjustment for multiple comparisons. Significant differences at the *p* < 0.01 level were observed for the pre-interview condition, post-interpersonal conversation, and post-conversation with the AI agent conditions, all compared to the condition after the MMSE.

### 6.2. Analysis of the STAI

The State-Trait Anxiety Inventory (STAI) is designed with a correlation of about 0.27 between trait anxiety and state anxiety. As in the VAS, a Friedman test was conducted in Microsoft Excel (Version 2404) to assess state anxiety in the moment. The *p*-value was 2.02 × 10^−10^, indicating that the STAI scores changed with test administration. As with the VAS, the post-hoc, two-group test was conducted with Bonferroni’s adjustment for multiple comparisons. Only after the MMSE were there significant differences at the *p* < 0.01 level for the pre-interview state, post-interpersonal conversation, and post-conversation with the AI agent. Correlation coefficients were calculated for state anxiety at the beginning of the experiment, state anxiety after the in-person conversation, state anxiety after the conversation with the AI agent, and state anxiety after the MMSE (Table 3).

**Figure 5:**
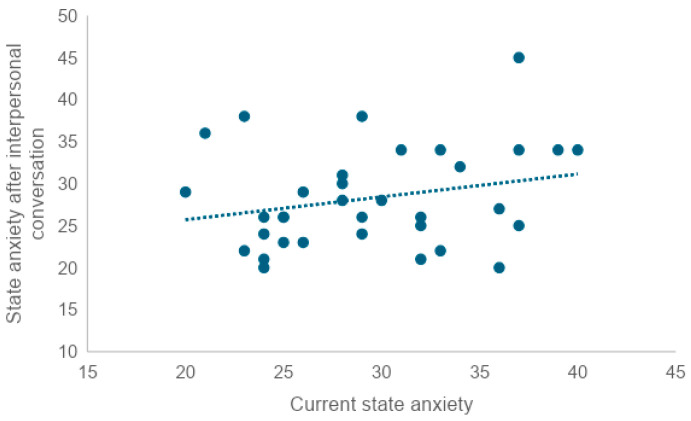
Correlation between current state anxiety and post-interpersonal conversation state anxiety (*n* = 34). The X-axis represents current state anxiety, while the Y-axis represents state anxiety after interpersonal conversation. Each point indicates individual participant data, and the dashed line shows the correlation between the two variables. The upward slope of the dashed trendline suggests that higher current state anxiety is associated with higher state anxiety after interpersonal conversation.

**Figure 6:**
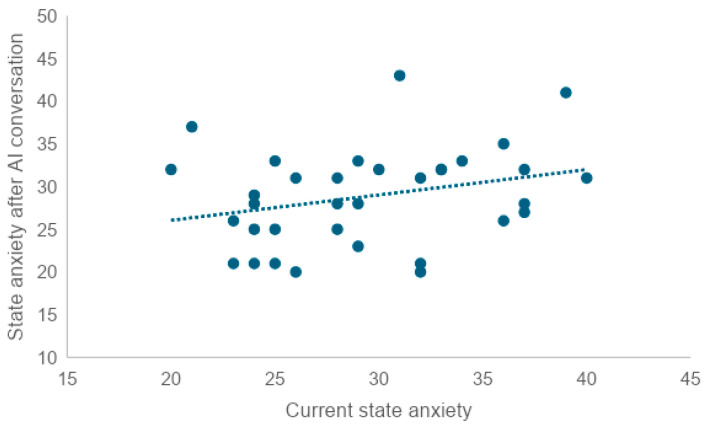
Correlation between current state anxiety and state anxiety after AI conversation (*n* = 34). The X-axis represents current state anxiety, while the Y-axis represents state anxiety after AI conversation. Each point shows individual participant data, and the dashed line indicates the correlation between the two variables. The upward trendline suggests that higher current state anxiety is associated with higher state anxiety after AI conversation.

**Figure 7:**
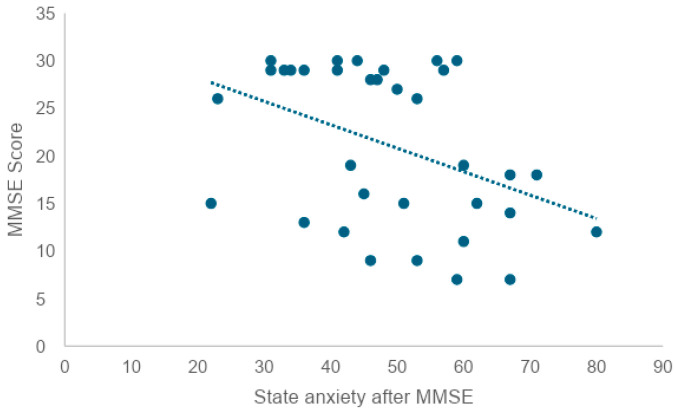
Correlation between post-MMSE state anxiety and MMSE scores (*n* = 34). The X-axis represents state anxiety after MMSE, while the Y-axis represents MMSE score. Each point shows individual participant data, and the dashed line indicates the correlation between the two variables. The downward trendline suggests that higher state anxiety after MMSE is associated with lower MMSE scores. The consistent direction of the points also indicates a potential negative correlation.

Scores slightly decreased after the in-person conversation and after the conversation with the AI agent. On the other hand, the mean increased after the MMSE. There was a very weak correlation between pre-experimental state anxiety and state anxiety after in-person and AI agent conversations, respectively (Rr = 0.25, R = 0.28). No correlation was found between pre-experimental state anxiety and post-MMSE state anxiety (R = 0.08) (Figure 5 and Figure 6). To examine whether there was a correlation between the state anxiety score after the MMSE and the MMSE score, the correlation coefficient was tested, revealing a weak correlation (r = −0.41) (Figure 7).

### 6.3. Analysis of ECG

In this study, we prioritized minimizing the burden on participants and used a Holter electrocardiograph. Bradycardia is generally defined as a resting heart rate of fewer than 60 beats per minute in adults [66], and some studies use a threshold of 50 beats per minute, considering the decrease in heart rate due to aging [67]. In Japan, the guidelines of the Japanese Circulation Society state that a heart rate of 40 beats per minute or less may warrant consideration of a pacemaker [68]. Based on these criteria, data indicating a heart rate of fewer than 40 beats per minute were considered outliers. The analysis was conducted using raw data with outliers excluded, and the average values for each session were analyzed.

**Figure 8:**
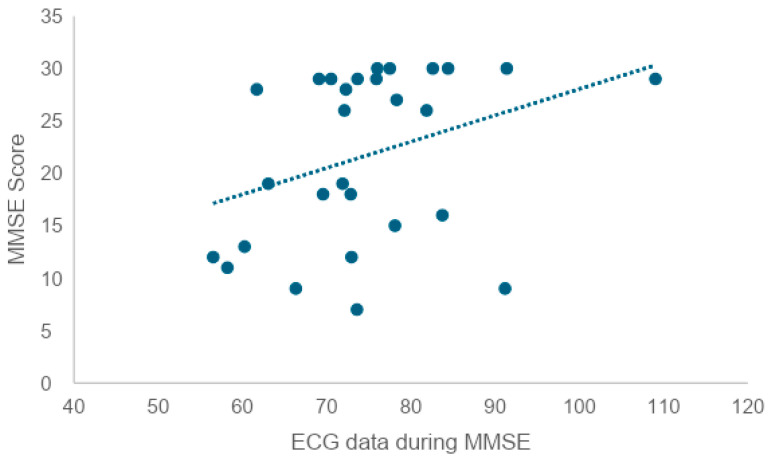
Correlation between ECG during MMSE and MMSE scores (*n* = 34). The X-axis represents ECG data during MMSE, while the Y-axis represents MMSE score. Each point shows individual participant data, and the dashed line indicates the correlation between the two variables. The upward trendline suggests that higher ECG data during MMSE is associated with higher MMSE scores.

To examine whether there is a correlation between heart rate during the MMSE and MMSE scores, we tested the correlation coefficient and found a weak correlation (r = 0.34), as shown in Figure 8.

There was also little correlation between the ECG data during the interpersonal conversation and the VAS and STAI scores after the interpersonal conversation (VAS r = 0.24, STAI r = 0.21) (Table 4). Similarly, there was little correlation between the ECG data during the conversation with the AI and the VAS and STAI scores after the conversation with the AI (VAS r = 0.18, STAI r = −0.07) (Table 5).

### 6.4. Relationship between Personality Traits and Each Assessment Item

**Figure 9:**
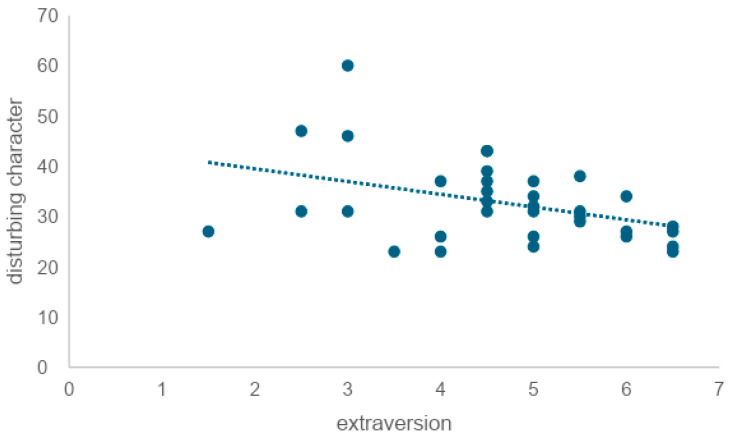
Correlation between extraversion and trait anxiety (*n* = 34). The X-axis represents extraversion, while the Y-axis represents disturbing character. Each point shows individual participant data, and the dashed line indicates the correlation between the two variables. The downward trendline suggests that higher extraversion is associated with lower disturbing character ratings.

The Japanese version of the Ten Item Personality Inventory (TIPI-J) [69] was used to measure the participants’ personality traits. The correlation coefficients between extraversion and VAS (r = −0.06) and STAI (r = −0.32) after conversing with a human showed no significant correlations. Similarly, to examine whether extraversion was correlated with VAS and STAI after the AI conversation, we tested the correlation coefficients and found no correlation between extraversion and either VAS (r = −0.21) or STAI (r = −0.28) after AI conversation. Next, to examine whether there was a correlation between extraversion measured by the TIPI-J and trait anxiety measured by the STAI, we tested the correlation coefficient and found a weak negative correlation (r = −0.39) (Figure 9).

## 7. Discussion

### 7.1. VAS and STAI Scores

Analyses of the VAS and STAI scores revealed that the mental burden of the MMSE was significantly higher than that of the other sessions. However, no significant differences were found between the baseline condition and the in-person and AI conversations. It was confirmed that cognitive function estimation based on spontaneous speech, as in previous studies, is preferable to formal evaluation tests such as the MMSE in order to reduce the psychological burden on the participants.

In communication with chatbots that provide emotional support, whether the message sender is human or a chatbot has been found to be a significant factor, with studies indicating that messages from humans are more effective [70]. However, in this study, it was demonstrated that the psychological burden experienced by participants was similar whether they were interacting with the AI agent system developed for this research or conversing with real humans. In the future, it may be possible to easily check cognitive function in the homes of elderly individuals without visiting facilities.

### 7.2. Analysis of STAI

In order to examine whether there is a correlation between trait anxiety and current state anxiety, state anxiety after an interpersonal conversation, state anxiety after an AI conversation, or state anxiety after MMSE administration, the correlation coefficient was tested. A weak correlation was found between trait anxiety scores and current state anxiety scores (r = 0.47). A very weak correlation was also found between trait anxiety and scores after an interpersonal conversation (r = 0.35), and between trait anxiety and scores after an AI conversation (r = 0.36). No correlation was found between trait anxiety and post-MMSE scores (r = 0.00).

To examine whether there was a correlation between current state anxiety and state anxiety after the in-person conversation, state anxiety after the AI conversation, and state anxiety after the MMSE, we tested the correlation coefficient. We found a very weak correlation between current state anxiety and scores after the in-person conversation (r = 0.25), as well as a very weak correlation between current state anxiety and scores after the AI conversation (r = 0.28). No correlation was found between trait anxiety and post-MMSE scores (r = 0.08).

A high level of current state anxiety or trait anxiety was associated with a similarly high level of the other. At the same time, neither current state anxiety nor trait anxiety was correlated with post-MMSE state anxiety. This suggests that neither state anxiety in the pre-MMSE situation nor anxiety as a relatively constant personality trait may have an effect on the STAI scores after the MMSE. On the other hand, since both current trait anxiety and current state anxiety showed a very weak correlation between state anxiety scores after the in-person conversation and after the AI conversation, anxiety as a personality trait and state anxiety in the pre-implementation situation may influence the degree of anxiety caused by the in-person conversation and AI conversation.

In addition, since there was a correlation between trait anxiety and current state anxiety, it is thought that when anxiety as a personality trait is high, anxiety under the situation is also high. Given this, an approach that reduces state anxiety before the MMSE may not necessarily reduce trait anxiety. In order to examine whether there is a correlation between the state anxiety score after the MMSE and the MMSE score, a correlation coefficient test revealed a weak negative correlation (r = −0.41). This suggests that the higher the state anxiety score after the MMSE, the lower the MMSE score. At the same time, it can also be interpreted that individuals with lower cognitive function tend to feel a greater psychological burden from being tested for cognitive function.

### 7.3. Personality Traits

To examine whether extraversion is correlated with trait anxiety, we tested the correlation coefficient and found a weak negative correlation (r = −0.39). This indicated that the lower the level of extraversion, the higher the level of trait anxiety. It is also reported that extraversion tends to be outwardly oriented in interests and that it favors socializing with a wide range of people and engaging in cheerful banter with fluent eloquence and skillful wit [69]. There was no correlation between extraversion and either VAS (r = −0.06) or STAI (r = −0.32) after in-person conversation, nor between VAS (r = −0.21) or STAI (r = −0.28) after AI conversation. Mou et al. (2017) pointed out that when interacting with AI, human users exhibit lower levels of openness, agreeableness, extraversion, conscientiousness, and self-disclosure compared to conversations with humans, indicating different personality traits and communication attributes in human–AI interactions [51]. However, this study suggested that extraversion might not be affected by whether the conversational partner is a human or an AI in everyday conversational situations.

### 7.4. Future Work

Further research is needed to determine whether the situation that was not significant in this analysis also holds for other age groups besides the elderly. The content of the responses of elderly participants may vary depending on whether the questioner is an AI agent or a live person (e.g., length and qualitative aspects of the responses). Further analysis of the transcribed text data is necessary to gain insights into the trends and characteristics of the responses.

In this study, no significant differences were found in the MMSE and STAI results. Therefore, it will be necessary in the future to conduct more detailed analyses, such as analyzing voice data obtained from speech and conducting group work after the experiment to gather feedback.

AI agents have the potential to assess cognitive function and levels of the Instrumental Activities of Daily Living (IADL) through dialogue with elderly individuals using natural language processing technologies. IADL refers to the activities necessary for an individual to live independently, and it is particularly essential for measuring the level of independence in daily life among the elderly. This method could provide a non-invasive and efficient alternative to traditional face-to-face assessments, especially in situations where visits are difficult.

Furthermore, there is potential for designing personalized care plans for each elderly individual based on data collected by AI agents. The International Classification of Functioning, Disability, and Health (ICF) provides a comprehensive framework for evaluating an individual’s health status and the associated levels of disability and social participation. It is expected to play a crucial role in assessing the overall picture of a person’s abilities and disabilities, especially in cognitive function evaluations for the elderly. Such an approach could not only improve the quality of life for elderly individuals who need support to continue living at home but also reduce the burden on caregivers and healthcare professionals.

## 8. Limitations

The dialogue engine used in this study sometimes stopped expanding on certain words, such as war, when it deemed them inappropriate. The challenge is to enable dialogue even with such word choices. Specifically, the engine could not provide appropriate responses to specific words, such as news about a murder case. In the future, it will be necessary to address this issue by improving the engine so that it can continue dialogue even when specific words are included.

In this study, the subjects were limited to elderly persons living in the community, and the conversation style was semi-structured interviews. Different results could be obtained with the elderly residing in nursing homes or hospitals or with a different conversational style. In Japan, it is difficult to select elderly people with cognitive decline as research subjects, and the sample size was small for this experiment. However, verification by prototype is necessary under such circumstances, and therein lies the contribution of this study. In the future, a follow-up study with a larger sample size is needed, using this study as a starting point.

## 9. Conclusions

This study investigated the psychological burden of various conversational formats, including interaction with an AI agent, on elderly participants during cognitive function assessment. Thirty-four participants (12 males and 22 females) with a mean age of 78.71 years were evaluated using the Mini-Mental State Examination (MMSE), the Visual Analogue Scale (VAS), and the State-Trait Anxiety Inventory (STAI). The study aimed to compare the psychological impact of conversational interaction (with both humans and AI agents) and cognitive testing on the participants. The results showed that mental strain, as measured by VAS and STAI scores, was significantly higher during the MMSE sessions compared to the other conversational formats (*p* < 0.01).

Interestingly, there was no significant difference in the mental burden between conversations with humans and AI agents. This suggests that AI-based conversational systems may be as effective as human interaction in cognitive assessments, potentially providing a less burdensome alternative for the elderly in various settings, including home environments. This finding underscores the importance of considering the psychological burden of cognitive assessment and highlights the potential of AI to reduce this burden.

## Figures and Tables

**Table 1:** Daily conversation used in interpersonal and agent conversations, consisting of 30 questions in 5 areas.

**(1) Process before coming to the hospital** Q1. Where is your home? Q2. How long did it take you to get here today? Q3. After you left your home, how did you come here? Q4. What time did you leave home to come to the hospital today?
**(2) Life history** Q5. Where were you bom? Q6. Do you have any siblings (if so, how many)? Q7. Which elementary school did you attend? Q8. What did you do after elementary school? (Which junior high school did you attend?) Q9. What did you do after graduating junior high school? (Which high school did you attend?) Q10. What do you do for work? (Do you have any memorable stories?) Q11. Are you married? (When was your wedding?) Q12. Do you have any children? (Where do your children live?)
**(3) Normal life** Q13. How do you usually spend your time? (Please tell us your approximate weekly schedule) Q14. What time do you get up in the moning and go to bed? Q15. How often do you go out? (Where do you go most often?) Q16. Do you bathe every day? (Do you bathe in a bathtub?) Q17. How do you prepare your meals? (Do you eat three meals a day?)/What did you eat last night? Q18. How do you clean your house? (How often do you dean your house?) Q19. How do you do your laudry? (How often do you do it?)
**(4) Interests** Q20. What news have you been interested in on TV or the Internet recently? Q21. Please tell me about a sad event that happened to you recently. Q22. Please tell me about a recent usettling event. Q23. Tell me about a recent event that made you angry. Q24. Tell me ab out a recent event that made you feel bad. Q25. Tell me about a recent event th at surprised you. Q26. Tell me ab out a recent happy event that happened to you. When did it happen? Q27. Tell me ab out someone you admire. Q28. What are you passionate about these days?
**(5) Plans for the rest of the day** Q29. What are your plans for the rest of the day? (How will you get home?) Q30. When was the date of your last visit?

**Table 2:** Comparison of differences in means by VAS and STAI.

				VAS				STAI			
	Age	GDS-15 Score	MMSE Score	Before Intervention	Human Conversation	AI Agent Conversation	MMSE	Before Intervention	Human Conversation	AI Agent Conversation	MMSE
Mean	78.71	2.48	21.09	5.50	7.15	9.76	42.88	29.41	28.26	28.85	48.85
SD	6.77	2.45	8.26	10.56	12.32	14.42	29.13	5.45	5.98	5.71	13.85

**Table 3:** Comparison of correlation coefficients between state anxiety before the experiment, after in-person conversation, after AI conversation, and after MMSE implementation.

	Mean	SD	r
State anxiety before the start of the experiment	29.41	5.37	―
After interpersonal conversation	28.26	5.89	0.25
After conversation with AI	28.85	5.63	0.28
After MMSE implementation	48.85	13.64	0.08

**Table 4:** Comparison of correlation coefficients between ECG data during interpersonal conversation and VAS and STAI after interpersonal conversation.

	Mean	SD	r
ECG during interpersonal conversation	75.27	10.25	—
VAS score after interpersonal conversation	7.15	12.14	0.24
STAI score after interpersonal conversation	28.26	5.89	0.21

**Table 5:** Comparison of correlation coefficients between ECG data during AI conversation and VAS and STAI after AI conversation.

	Mean	SD	r
ECG during AI conversation	74.81	11.21	—
VAS score after AI conversation	9.76	14.21	0.18
STAI score after AI conversation	28.85	5.63	−0.07

## Data Availability

Based on the requirements for the ethical review and the protocols outlined by our University for storing and sharing data, our data, which includes information on dementia patients, will be disclosed upon reasonable request.
